# Impact of tobacco control auxiliary resources on the 5As behavior in nursing interns: Self-reports from students

**DOI:** 10.18332/tid/125231

**Published:** 2020-08-05

**Authors:** Li Zhang, Jun Li, Yalan Lv, Xia Yang, Li Bai, Yetao Luo, Yanhan Chen, Yong Zhao

**Affiliations:** 1 College of Nursing, Chongqing Medical University, Chongqing, China; 2 The Office of Chongqing Medical University, Chongqing, China; 3 College of Medical Informatics, Chongqing Medical University, Chongqing, China; 4 Department of Clinical Epidemiology and Biostatistics, Children’s Hospital, Chongqing Medical University, Chongqing, China; 5 School of Public Health and Health Management, Chongqing Medical University, Chongqing, China

**Keywords:** tobacco cessation, 5As, nursing interns, tobacco control resources, tobacco control auxiliary tools

## Abstract

**INTRODUCTION:**

The help of healthcare professionals for smokers to quit is critically important to increase quit rates. In the future, internship nursing students will potentially become the largest population of medical professionals. This study explored the impact of the use and awareness of universal tobacco control auxiliary resources on nursing students’ 5As behavior in helping patients to quit smoking during a 40-week clinical internship in the last year of nursing school in Chongqing, China.

**METHODS:**

A survey was conducted in 13 teaching hospitals selected from 29 in Chongqing, China, in 2019, by a random cluster sampling method. It investigated, by self-reported questionnaires, student 5As behavior in helping patients to quit smoking and use and knowledge of tobacco cessation auxiliary resources (tobacco cessation self-education manual, tobacco cessation guidelines, tobacco cessation drugs, tobacco cessation websites, and hotline). The relationship between tobacco cessation auxiliary resources and 5As behavior in helping patients to quit smoking was analyzed with a multivariate linear mixed-effect model.

**RESULTS:**

In all, 534 (39.3%) students, of a total of 1358, reported that the majority of internship units provided a tobacco control self-education manual; 674 (49.6%) ever skimmed through tobacco cessation guidelines; 641 (47.2%) browsed tobacco cessation websites; 738 (54.3%) knew some cessation-assistance drugs; and 93 (6.8%) knew of and could recall the tobacco cessation hotline. Except for tobacco cessation websites, tobacco cessation auxiliary resources affected nursing interns’ 5As behavior in helping patients to quit (p<0.05).

**CONCLUSIONS:**

Tobacco cessation auxiliary resources influenced students’ 5As behavior in helping patients to quit smoking. Students knew a little of tobacco cessation auxiliary resources. To improve students’ 5As behavior for helping patients to quit, more tobacco cessation resources need to be developed and more students need to become acquainted with them.

## INTRODUCTION

China is the largest producer and consumer of tobacco in the world. More than one million Chinese die each year from tobacco-related diseases^1^. China will be confronted with a larger number of premature deaths if no rapid, extensive and effective measures are takento decrease smoking rates^2^. Short interventions for smoking cessation from healthcare professionals can help smokers to enhance their intention to quit^3^, which is significant for smoking control. The World Health Organisation (WHO) recommends that all healthcare professionals, including physicians, dentists, pharmacists, nurses and others, assume the responsibility to help smokers quit^4^. China Health and Family Planning Commission take active measures to support smoking cessation, including the establishment of smoking cessation clinics^5^, and the development of the *Smoking Cessation Guidelines* ^6^ advising healthcare professionals to administer short interventions of tobacco cessation in patients, such as recording smoking history, giving advice to quit, assessing degree of tobacco dependence and willingness to quit, offering help in quitting, and arranging follow-up services. Some hospitals use Healthcare Information System (HIS) to support healthcare professionals to help smokers quit smoking^7^. However, many smokers seldom receive quitting suggestions or support when they visit doctors, and their smoking histories are rarely inquired into^8^. Only when patients’ disease and smoking are linked, or smoking-related disease symptoms develop, do doctors discuss the smoking problem and proceed to help them to quit^9^. But exceptions do exist. Recently, a European transnational survey revealed that doctors felt frustrated with smoking patients who were undergoing chronic obstructive pulmonary disease (COPD). These frustrations hindered the provision of evidence-based treatment for smoking cessation^10^. It seems that healthcare professionals neglect the duty to assist with tobacco cessation^11^. It appears that many factors, internal as well as external, result in healthcare professionals not helping smokers to quit.

Obstacles for medical professionals to help smokers quit include lack of time, lack of health insurance, and supportive circumstances^9,12,13^. Also, healthcare professionals are not likely to provide smoking cessation interventions when they detect no smoking cessation related requirements, no intention to quit, no desire for professional help by the patient, or when assistance with smoking cessation is deemed to impair their relationship with the patient^9,14,15^. Healthcare professionals’ outcome expectations, attitudes, self-efficacy, training experience, knowledge or skills of tobacco cessation and their own smoking histories all impact on the help patients receive to quit smoking^9,12,15-21^.

Different professional specialities lead to varying behaviours in helping smokers to quit^13,22^. Nurses play significant roles in health maintenance and promotion and are found to be functioning in smoking cessation^23^. In the Netherlands, nurses, compared with gynecologists or pediatricians, encountered fewer obstacles in advocating tobacco cessation^13^. Smoking prevention and reduction were found to be more suitable for the work of professional nurses^13^, but limited education and training hindered them from providing a better tobacco cessation service^24^.

Nursing students are potentially future nurses so their behaviour in helping smokers quit during their 9-month (40 weeks) clinical internship in the last year of nursing school affects future interventions in their nursing careers. The main hindrances stopping nursing students from helping smokers to quit are low self-efficacy for tobacco cessation support, smokers themselves, no motivation from patients, lack of training or knowledge, and a lack of communication skills^25,26^. In addition, the effects of tobacco cessation auxiliary resources on the behaviour of nursing students in helping patients to quit remain relatively unknown. Thus, this study explored the potential of these resources in facilitating nursing students’ behaviour towards providing 5As smoking cessation to patients during their clinical internship.

## METHODS

### Study design

The design and methods of this cross-sectional study in Chongqing have been published previously^26^. An online survey was conducted in nursing students from 13 teaching hospitals in order to understand their 5As behaviour and interfering factors in helping smokers quit during clinical internship. The study was approved by the Ethics Committee at the First Affiliated Hospital of Chongqing Medical University (2019-157). The participating nursing students were in clinical internship in their last year before graduation. The study involved 1522 students from a response rate of 62.4% (1522/2400). Valid questionnaires were 1358, corresponding to an acceptance rate of 89.2%, after removing those who finished in less than 180 seconds or addressed non-Chongqing areas.

### Questionnaire

#### Demographic, attitude and self-efficacy characteristics

Demographic characteristics included gender, age, education, clinical internship duration and whether a current smoker. The 30 tobacco-control knowledge questions (4 tobacco control resources were removed from the original 34 of the reported study^26^) were scored by the percentage of correct responses with respondents divided into high and low score groups based on the median score. Attitudes and self-efficacy about participating in tobacco control were assessed by the items: 1) healthcare professionals should routinely perform 5As to provide smokers brief smoking cessation interventions; 2) I have received enough training about tobacco cessation; and 3) I am confident in helping smokers quit. Response options were evaluated on a 1–5 scale with: 1=Strongly disagree; 2=Disagree; 3=Neutral; 4=Agree; and 5=Strongly agree. These were then recoded into: 1–2 as Disagree; 3 as Neutral; and 4–5 as Agree.

#### Tobacco control related resources

Students responded to the questions on universal tobacco control auxiliary resources in China. The resources were: tobacco control self-education manual, tobacco cessation pharmacotherapy, tobacco cessation hotline, tobacco control websites, and the *China Tobacco Cessation Guidelines*. Questions were: 1) What is the distribution of health education materials about instructing patients to quit smoking in your internship units? (responses: 1=none, 2=rarely, 3=half of the units, 4=three-quarters of the units, and 5=all of the units); 2) Do you know about pharmacotherapy for helping patients quit smoking? (responses: 1=I don’t know, 2=I’ve heard of them but don’t know, 3=I’ve read the instructions, 4=I’ve introduced them to smokers, and 5=I’ve instructed smokers to use them; these were recoded as: 1–2 Don’t know, 3 Know about a few, 4–5 Know well); 3) What is your knowledge about the tobacco cessation hotline? (responses: 1=Know and can recall it correctly, 2=Heard of it but cannot recall correctly, and 3=Don’t know); 4) Do you know the *China Clinical Tobacco Cessation Guidelines* and tobacco cessation websites? (responses: 1=I don’t know, 2= I’ve heard of them, 3=I’ve surfed, 4=I’m familiar with them, and 5=I’m already completely familiar with them; these were recoded: 1–2 Don’t know, 3–5 Have scanned).

#### The 5As behaviour score for helping patients

Students answered questions about 5As behaviours in helping patients to stop smoking during their internship. The 5As are : Ask patients about their smoking; Advise smokers explicitly to quit smoking; Assess smokers desire to stop smoking; Assist smokers to quit smoking; and Arrange follow-up services for the smokers who wish to initiate quitting. Each 5As behaviour was scored on a scale of 1–5, based on the number of patients helped: 1 = none, 2 = 1–3, 3 = 4–9, 4 = 10–25, and 5 >25 patients. The summed behaviour score ranged from 5 to 25.

### Statistical analysis

Data were analyzed with the statistical software package SPSS20.0 (IBM Corporation, Armonk, NY, USA) and characteristics of the participants were introduced by descriptive analysis and summarized with frequency and percentage. Taking hospitals as cluster units, a multi-factor mixed-effect model was employed to study the components influencing the 5As behaviour of students in helping smokers to quit. The multi-factor analysis covered basic characteristics, knowledge of tobacco control, attitude and self-efficacy of tobacco control, and use and knowledge of tobacco control auxiliary resources. A p<0.05 was considered statistically significant in the model.

## RESULTS

### 5As behaviour in helping patients

Among the 1358 nursing students, 1243 (91.5%) ever asked patients about smoking; 1148 (84.5%) advised smokers to quit; 1115 (82.1%) assessed patients’ intention to stop smoking; 1080 (79.5%) offered assistance to smokers; and 974 (71.7%) arranged follow-up services in the cessation processing (Table 1).

**Table 1:** 5As behaviour frequency of helping patients in nursing interns’ practice in 2019 in Chongqing, China (N=1358)

*Number of patients helped*	*Ask*		*Advice*		*Assess*		*Assist*		*Arrange*	

	*n*	*%*	*n*	*%*	*n*	*%*	*n*	*%*	*n*	*%*
0	115	8.5	210	15.5	243	17.9	278	20.5	384	28.3
1–3	378	27.8	430	31.7	435	32.0	436	32.1	397	29.2
4–9	344	25.3	361	26.6	348	25.6	343	25.3	313	23.0
10–25	236	17.4	201	14.8	189	13.9	174	12.8	147	10.8
>25	285	21.0	156	11.5	143	10.5	127	9.4	117	8.6

### Knowledge of tobacco control auxiliary resources and their influence on students’ 5As behaviour to help patients

In all, 574 students (42.3%), among 1358, reported that their internship units provided no or few self-help materials to help patients quit smoking. There were: 684 (50.4%) who did not know the *China Clinical Tobacco Control Guidelines*; 717 (52.8%) did not know tobacco control websites; 620 (45.7%) did not know about tobacco cessation pharmacotherapy; 93 (6.8%) knew and could recall the tobacco cessation hotline correctly; 626 (46.1%) had heard of the hotline but could not recall it correctly; and 639 (47.1%) responded with ‘don't know’. All the tobacco control resources affected students’ 5As behaviour (Table 2).

**Table 2:** The relationship between nursing interns’ knowledge of tobacco control resources and 5As score in 2019 in Chongqing, China (N=1358)

*Knowledge*			*All students*	

	*n*	*%*	*Median (P25, P75)*	*p*
**Self-help materials of tobacco cessation for health education had by internship departments**				<0.001
None/seldom	574	42.3	10.0 (7.0, 13.0)	
Half of the departments	250	18.4	14.0 (10.0, 16.0)	
Most	534	39.3	16.0 (13.0, 20.0)	
**The China Clinical Tobacco Control Guidelines**				<0.001
Don’t know	684	50.4	10.0 (8.0, 15.0)	
Have scanned	553	40.7	15.0 (10.0, 18.0)	
Know well	121	8.9	20.0 (15.0, 25.0)	
**Tobacco cessation websites**				<0.001
Don’t know	717	52.8	10.0 (8.0, 15.0)	
Have scanned	528	38.9	15.0 (10.0, 19.0)	
Know well	113	8.3	20.0 (15.0, 25.0)	
**Pharmacotherapy that helps patients to quit smoking**				<0.001
Don’t know	620	45.7	10.0 (8.0, 15.0)	
Know about a few	598	44.0	15.0 (10.0, 18.0)	
Know well	140	10.3	20.0 (15.0, 25.0)	
**Tobacco cessation hotline**				<0.001
Don’t know	639	47.1	10.0 (8.0, 15.0)	
Have heard of it but can’t recall correctly	626	46.1	15.0 (10.0, 19.0)	
Know and can recall it correctly	93	6.8	20.0 (15.0, 24.0)	

### The relationship between tobacco control knowledge, attitude, self-efficacy and 5As behaviour

A total of 765 (56.3%) students thought healthcare professionals should apply 5As routinely to provide tobacco cessation services for patients; 578 (42.6%) believed receiving enough education on tobacco cessation, and 290 (21.4%) were confident to help smokers quit. The average score on the 30 tobacco control knowledge questions was 0.760±0.144 with 706 (52.0%) scores over the median value of 0.8. Students’ tobacco control knowledge and attitude both had an effect on 5As behaviour (p<0.05) (Table 3).

**Table 3:** Effect of tobacco control attitude on nursing interns’ participation in tobacco control in 2019 in Chongqing, China (N=1358)

*Knowledge and Attitude*			*All students*	

	*n*	*%*	*Median (P25, P75)*	*p*
**Tobacco control knowledge** (median: 0.8)				<0.001
<0.8	652	48.0	12.0 (10.0, 16.0)	
≥0.8	706	52.0	14.0 (10.0, 18.0)	
**Healthcare professionals should use the 5As routinely to provide tobacco cessation services for patients**				<0.001
Disagree	56	4.1	10.0 (7.5, 13.5)	
Neutral	537	39.5	10.0 (9.00, 15.0)	
Agree	765	56.3	15.0 (10.0, 20.0)	
**I have received enough tobacco cessation intervention training**				<0.001
Disagree	214	15.8	10.0 (8.0, 13.0)	
Neutral	566	41.7	11.0 (10.0, 15.0)	
Agree	578	42.6	15.0 (10.0, 20.0)	
**My confidence to help smokers quit smoking** (self-efficacy)				<0.001
Self-distrust	202	14.9	10.0 (8.0, 15.0)	
Neutral	866	63.8	12.0 (10.0, 16.0)	
Self-trust	290	21.4	16.0 (12.0, 20.0)	

### Multi-factor analysis of 5As behaviour

A multi-factor analysis of 5As behaviour was conducted taking the 5As summed score as the dependent variable, and independent variables the basic characteristics, self-reporting knowledge of tobacco control, attitude and self-efficacy of tobacco control, with the hospital as cluster unit. The analysis revealed that the factors affecting the implementation of 5As behaviour were: tobacco control knowledge, healthcare professionals’ sense of responsibility, and enough training on tobacco control. Except for knowledge about tobacco control websites, tobacco control auxiliary resources such as the distribution of tobacco control self-help materials in the internship units, scanning tobacco control guidelines, being acquainted with tobacco control pharmacotherapy, knowing and recalling the tobacco control hotline, all had an effect on students’ 5As behaviour (Table 4).

**Table 4:** Analysis by multivariate and mixed-effect model on 5As of nursing interns of smoking cessation, tobacco cessation resources and nursing interns’ primary characteristics in 2019 in Chongqing, China (N=1358)

*Variables*	*Estimation*	*SE*	*p*	*95% CI*	
				*Lower*	*Upper*
**Gender**					
Female	Ref.				
Male	0.023	0.482	0.963	-0.922	0.968
**Age** (years)					
≤17	Ref.				
18	-0.197	0.482	0.683	-1.142	0.748
19	0.881	0.643	0.171	-0.380	2.142
20	-0.025	0.571	0.964	-1.145	1.094
21	0.296	0.592	0.617	-0.864	1.457
22	-0.321	0.635	0.613	-1.567	0.925
≥23	-0.029	0.727	0.968	-1.455	1.397
**Professional education**					
Diploma	Ref.				
Assoc. degree	-0.528	0.501	0.292	-1.511	0.454
Baccalaureate	-0.183	0.649	0.778	-1.458	1.092
**Current smoker**					
No	Ref.				
Yes	-0.372	0.782	0.635	-1.906	1.162
**Internship time** (months)					
4	Ref.				
5	0.903	0.638	0.157	-0.349	2.155
6	1.395	0.654	0.033	0.111	2.679
7	1.155	0.727	0.112	-0.272	2.582
8	1.475	0.823	0.073	-0.139	3.089
**Tobacco knowledge score**					
<0.8	Ref.				
≥0.8	0.518	0.247	0.036*	0.033	1.003
**Healthcare professionals routinely use 5As to treat smokers**					
Disagree	Ref.				
Neutral	0.488	0.629	0.437	0.745	1.722
Agree	1.522	0.628	0.016*	0.290	2.755
**I have received enough tobacco control training**					
Disagree	Ref.				
Neutral	0.277	0.373	0.457	-0.454	1.008
Agree	1.174	0.402	0.004*	0.386	1.961
**My confidence to help smokers quit smoking**					
Self-distrust	Ref.				
Neutral	-0.292	0.357	0.413	-0.992	0.408
Self-trust	0.765	0.453	0.091	-0.123	1.653
**Self-help materials of tobacco cessation for health education had by internship departments**					
None/seldom	Ref.				
Half of the departments	1.496	0.347	0.000**	0.815	2.178
Most	3.716	0.307	0.000**	3.113	4.318
**The China Clinical Tobacco Control Guidelines**					
Don't know	Ref.				
Have scanned	1.187	0.373	0.002*	0.455	1.919
**Tobacco control websites**					
Don't know	Ref.				
Have scanned	-0.027	0.412	0.947	-0.835	0.780
**Pharmacotherapy that helps patients to quit smoking**					
Don't know	Ref.				
Know about a few	1.200	0.346	0.001*	0.521	1.879
Know well	3.398	0.539	0.000**	2.340	4.456
**Tobacco cessation hotline**					
Don't know	Ref.				
Have heard of it but can’t recall correctly	0.462	0.300	0.123	-0.126	1.050
Know and can recall it correctly	1.535	0.559	0.006*	0.439	2.631

* p<0.05

** p<0.001 (statistically significant). We have adjusted hospitals.

## DISCUSSION

We found that tobacco control auxiliary resources have an impact on nursing students’ 5As behaviour in helping smokers to quit. The survey revealed that students knew little about tobacco control auxiliary resources, which influenced students’ tobacco control behaviour to some extent. Therefore, in the process of guiding students to take part in tobacco control, more tobacco control auxiliary resources, in addition to the development of knowledge and skills, need to be provided to increase 5As behaviour. Nevertheless, little is known about what resources are relevant to students’ tobacco control behaviour in helping patients. This study explored the auxiliary resources that influenced students’ tobacco control interventions.

Students who knew about tobacco cessation pharmacotherapy provided patients with more smoking cessation help. Smokers’ physiological dependence on tobacco nicotine is an obstacle for smokers to quit while smoking cessation pharmacotherapy can relieve withdrawal symptoms and discomfort. The tobacco cessation reality in China is that drug therapy alone or in combination with the smoking cessation interventions could enhance quit rates^27^, but pharmacotherapy is still a barrier for nursing staff in tobacco cessation services^28^. Gaining insight into pharmacotherapy administration can improve nursing students’ 5As behaviour in helping smokers quit, and tobacco cessation self-help materials are the most universal and straightforward tobacco control education resources in a clinic^29^. Although some have reported that self-help education material help smokers less than other resources^30^, nevertheless such convenient and accessible resources in internship units provide the possibility for students to improve their behaviour towards helping patients quit smoking. When smokers would like more tobacco cessation assistance, students can recommend a smoking control clinic or provide a tobacco cessation hotline. Hence, students who can recall a tobacco cessation hotline correctly can perform more 5As behaviour on patients and are more likely to recommend it to smokers. The *Clinical Smoking Cessation Guidelines* report advises students in detail on the steps and methods for helping smokers to quit, including strategies and resources. The report plays an active and positive guiding role for students to help smokers quit, with substantial flexibility. Tobacco control websites were found not to influence students’ 5As behaviour in helping patients, probably because the content on these sites is not tailored to students’ behaviour for helping patients quit smoking. Currently, the official website of China Tobacco Control Office involves massive and extensive professional content on the tobacco epidemic, tobacco hazards and tobacco control policies, and tobacco control requirements from various sections. However, the suggested few specific and targeted methods make them impractical to recommend as websites to smokers to help them quit. Consequently, tobacco control websites should maximize messages on tobacco control advantages and give updates on more operable and user-friendly resources, with more emphasis on user experiences.

As expected, more knowledge, adequate tobacco control education and positive attitudes of using 5As routinely to help smokers quit were related to students’ 5As behaviour. Students’ basic characteristics had no bearing on tobacco control 5As behaviour, consistent with previous findings^24^.

### Strengths and limitations

This study is the first survey to explore the impact of tobacco control auxiliary resources on nursing students’ 5As behaviour in helping patients quit smoking in China. It provides valuable information that can be applied to improve the 5As behaviour of nursing students in assisting smokers to quit. This study has some limitations. First, listed and examined tobacco control auxiliary resources covered only the universal ones in China, such as tobacco control manual (text), tobacco cessation guidelines, tobacco control websites, tobacco cessation hotline and tobacco cessation pharmacotherapy, but new media resources such as audio and video were not involved. For tobacco cessation pharmacotherapy, China currently prescribes mainly Varenicline and Bupropion and little nicotine replacement therapy (NRT). Moreover, 57% of smoking cessation clinics provide no smoking cessation pharmacotherapy, which conversely hinders students from helping patients to quit smoking^7^, while the China Guidelines do not recommend e-cigarette-assisted smoking cessation. Second, it is a cross-sectional survey and no causal inference of results can be made. Furthermore, the data were collected from self-reported questionnaires, which might result in bias because of social desirability. Third, the data were collected in Chongqing, and do not represent the national population. Therefore, a larger scale study is needed in the future.

## CONCLUSIONS

Tobacco control self-help materials that are easily accessible in the internship units and pharmacotherapy helping smokers overcome withdrawal symptoms have greater influence on 5As behaviour, followed by familiarity with the *Clinical Tobacco Control Guidelines* and smoking cessation hotline. To improve the 5As practice of students in helping patients quit smoking, it is urgent that relevant sections and departments develop and provide more tobacco control resources, in addition to knowledge and skills. *Clinic Tobacco Control Guidelines* and continuing education and training should underline the importance of smoking cessation methods and auxiliary resources, ensuring more healthcare professionals apply 5As behaviour to help smokers to quit.
